# Botulinum toxin A-induced muscle paralysis stimulates Hdac4 and differential miRNA expression

**DOI:** 10.1371/journal.pone.0207354

**Published:** 2018-11-14

**Authors:** Leah E. Worton, Edith M. Gardiner, Ronald Y. Kwon, Leah M. Downey, Brandon J. Ausk, Steven D. Bain, Ted S. Gross

**Affiliations:** Department of Orthopaedics and Sports Medicine, University of Washington, Seattle, WA; University of California, Davis, UNITED STATES

## Abstract

At sufficient dose, intramuscular injection of Botulinum toxin A causes muscle wasting that is physiologically consistent with surgical denervation and other types of neuromuscular dysfunction. The aim of this study was to clarify early molecular and micro-RNA alterations in skeletal muscle following Botulinum toxin A-induced muscle paralysis. Quadriceps were analyzed for changes in expression of micro- and messenger RNA and protein levels after a single injection of 0.4, 2 or 4U Botulinum toxin A (/100g body weight). After injection with 2.0U Botulinum toxin A, quadriceps exhibited significant reduction in muscle weight and increased levels of ubiquitin ligase proteins at 7, 14 and 28 days. Muscle *miR-1* and *miR-133a/b* levels were decreased at these time points, whereas a dose-responsive increase in *miR-206* expression at day 14 was observed. Expression of the *miR-133a/b* target genes *RhoA*, *Tgfb1* and *Ctfg*, and the *miR-1/206* target genes *Igf-1* and *Hdac4*, were upregulated at 28 days after Botulinum toxin A injection. Increased levels of Hdac4 protein were observed after injection, consistent with anticipated expression changes in direct and indirect Hdac4 target genes, such as *Myog*. Our results suggest Botulinum toxin A-induced denervation of muscle shares molecular characteristics with surgical denervation and other types of neuromuscular dysfunction, and implicates *miR-133*/Tgf-β1/Ctfg and *miR-1*/Hdac4/Myog signaling during the resultant muscle atrophy.

## Introduction

In addition to cosmetic applications, intramuscular injection of Botulinum toxin A (BoNT/A) has been used to treat a range of conditions with underlying muscle dystonia, including facial palsy, pain and muscle spasticity [1–4]. This neurotoxin acts by targeting SNARE protein synaptosomal-associated protein 25 (SNAP-25) for cleavage, which prevents the regulated secretion of neurotransmitter acetylcholine (Ach) at the neuromuscular junction (NMJ). Inhibition is transient, lasting about 3–4 months in humans and about 4 weeks in mice [3], and recovery involves synaptic remodeling of the NMJ. However, at sufficient dose, BoNT/A causes rapid muscle atrophy, a response that is conserved across species [5, 6].

Molecular events following BoNT/A intramuscular injection occur in stages, including an early response transcriptional adaptation with upregulation of genes encoding nicotinic acetylcholine receptor (nAChR) components and muscle specific tyrosine kinase receptor (Musk), responsible for clustering nAChRs [7, 8]. Intermediate transcriptional changes that follow the early responses are consistent both with muscle atrophy and with activation of muscle regeneration by myogenic transcription factors [7, 9]. Muscle atrophy following disuse, disease or denervation leads to an increase in catabolism of muscle proteins through cellular proteolysis [10], achieved with the increased expression of ubiquitin ligases *Atrogin-1* (Fbxo32; F-box only protein 32) and *MuRF-1* (Trim63; tripartite motif-containing 63), which are regulated by transcription factor Myogenin (Myog) and by class II histone deacetylases (Hdac) [11–14]. The role of ubiquitin-mediated proteolysis in BoNT/A-induced muscle atrophy is still ambiguous, but *Atrogin-1* and *MuRF-1* may be involved [9, 15]. Finally, transcriptional changes in response to BoNT/A injection establish remodeling of the extracellular matrix (ECM) with activation of regulators of collagen production [7] which is paralleled by changes in passive mechanical properties and elasticity of the muscle [5, 16]. While BoNT/A effects have generally been considered transient and reversible (e.g., versus surgical neurectomy [17]), there is increasing evidence that BoNT/A also has long term effects on muscle tissue and that these effects may be more apparent with multiple injections or at higher doses. At sufficient dose, BoNT/A reduction of muscle fiber area also alters the myosin heavy chain composition of muscle and stimulates fibrotic responses that may adversely affect the quality of recovered muscle tissue [18–24].

Recent studies, in part from our group, have revealed that BoNT/A-induced muscle paralysis (a single injection of 2.0U/100g in a single muscle) precipitates not only muscle atrophy, but also rapid and localized bone resorption (within the first two weeks following injection), and that this bone loss is minimally related to changes in skeletal loading due to altered gait [25–30]. These studies provide tissue level support for a growing literature emphasizing multiple levels of communication between muscle and bone [31]. In considering potential unidentified signaling pathways that might couple muscle atrophy and bone cell function, we were drawn to recent reports of miRNA mediation of neuronal dysfunction in muscle [32–34].

Small non-coding micro-RNA (miRNA) molecules regulate diverse cellular processes by sequence-specific targeting of messenger RNA transcripts for degradation or for suppression of translation. As might be expected, miRNAs have been found to mediate both muscle development and atrophy [35, 36]. About 25% of miRNA expressed in skeletal muscle consists of muscle specific species, including *miR-1*, *miR-133a/b* and *miR-206* [37], which exhibit elevated expression during muscle development [38]. *miR-1* and *miR-206* are closely related and share a seed sequence, and *miR-133a* and *miR-133b* differ by only a single nucleotide at their 3′ ends. *miR-1/206* and *miR-133* have distinctive effects in muscle development, with *miR-1/206* promoting differentiation by downregulation of targets such as *Hdac4* and gap junction protein alpha 1 (*Gja1*) in skeletal muscle [32, 39, 40], and *Mef2a* (myocyte enhancer factor 2A) in cardiac muscle [41]. *miR-133* has been reported both to promote myoblast proliferation by inhibiting expression of serum response factor (*Srf*) [39] which is required for both muscle proliferation and differentiation [42, 43] and also to inhibit myoblast proliferation through the ERK signaling pathway [44].

Given its widespread clinical use, the early molecular events and mRNA induction following intramuscular BoNT/A injection have been explored [7, 8, 45]. However, to our knowledge, altered expression of miRNA following transient BoNT/A-induced muscle paralysis has not been reported. As an initial exploration, we therefore assessed expression of miRNA and potential downstream targets in muscle following a single injection of BoNT/A.

## Materials and methods

### Animals and BoNT/A-induced denervation model

All animal experiments were performed using protocols and procedures approved by the Institutional Animal Care and Use Committee of the University of Washington. Female C57Bl/6 mice were obtained from Jackson Laboratories (Sacramento, CA, USA) and were 20–26 weeks of age at the initiation of the experiments. Transient muscle paralysis was induced in the right quadriceps muscle group via a single injection of botulinum neurotoxin A (BoNT/A) as described previously [25]. Quadriceps paralysis was confirmed 24 hours post-injection by visual examination of reduced extension of the affected limb [25, 26]. Animals were allowed free cage activity and food and water ad libitum throughout the experiment.

For miRNA/mRNA analyses, mice were randomized into three BoNT/A dosing groups (Units of BoNT/A/100g body weight) of 0.4U, 2.0U and 4.0U (n = 4-6/dosage), or untreated age-matched controls (Naïve, n = 10). For protein analyses, mice were randomized into a 2.0U BoNT/A dosage group and Naïve controls. This arm of the experiment was limited to a single BoNT/A dosage to minimize animal use. Mice from each group were then chosen at random for euthanasia on day 7, 14 or 28 and specimen collection as described below (n = 4-6/group/time point in BoNT/A, n = 10 Naïve controls). Day 28 was chosen as the final time point, as this was the point of maximal BoNT/A-induced muscle atrophy in our previous studies [26].

### RNA preparation and quantitative RT-PCR

Quadriceps muscles were collected into RNA*later* Solution (Ambion) and stored at 4°C until all experimental samples were collected (up to 28 days). A 25mg section of the mid belly of the quadriceps was cut for further processing. miRNA and mRNA were extracted from this muscle section using the miRNeasy Mini and RNeasy MinElute Cleanup kits (Qiagen). miRNA was analyzed using TaqMan Small RNA Assays (Applied Biosystems: catalog # 002222, 002246, 002247, 000510, 001973) and ViiA7 Real-Time PCR System (Applied Biosystems; Thermo Fisher). cDNA was synthesized from mRNA using Superscript III reverse transcriptase (Thermo Fisher), and analyzed by quantitative RT-PCR using SYBR green on the ViiA7. Gene expression levels relative to U6 small nuclear RNA and β-actin for miRNA and mRNA, respectively were quantitated using the 2^(-ΔΔCT) method. Canonical muscle *miR-1*, *-133a*, *-133b* and *-206* were investigated. Validated target genes of these muscle specific miRNAs (shown in Table 1) were chosen due to their reported roles in muscle. Primer sequences used in this study are listed in S1 Table.

**Table 1:** Validated muscle miRNA target genes.

miRNA	Target gene	Context
*miR-1/206*	*Pax3* (paired box 3)	Transcription factor downregulated to promote myogenesis [46]
	*Pax7* (paired box 7)	Transcription factor downregulated for differentiation of skeletal muscle satellite cells [47]
	*Hdac4* (histone deacetylase 4)	Transcriptional repressor of muscle gene expression [32, 39]
	*Gja1* (gap junction protein, alpha 1)	Gap junction protein regulated during myogenesis [40]
	*Igf-1* (insulin like growth factor 1)	Growth factor targeted during myocardial infarction; inhibits cardiomyocyte apoptosis [48, 49]
	*Met* (met proto-oncogene)	Tyrosine protein kinase upregulated in rhabdomyosarcoma [50]
	*Utrn* (utrophin)	Cytoskeletal protein downregulated during skeletal muscle differentiation [51]
*miR-133a/b*	*Srf* (serum response factor)	Transcription factor involved in myoblast proliferation and myogenesis [39]
	*RhoA* (ras homolog family member A)	Rho GTPase involved in cardiac hypertrophy [52, 53]
	*Tgfb1* (transforming growth factor beta 1)	Growth factor upregulated in atrial fibrosis [54]
	*Ctgf* (connective tissue growth factor)	Growth factor targeted in bladder smooth muscle cell fibrosis; fibrosis of cardiac myocytes [55, 56]
	*Egfr* (epidermal growth factor receptor)	Growth factor receptor targeted in prostate cancer cells [57]
	*Gja1* (gap junction protein, alpha 1)	Gap junction protein regulated during heart regeneration in zebrafish [58]

### Immunoblotting and antibodies

Left and right quadriceps muscles were weighed and then snap frozen in liquid nitrogen. Samples were ground to a powder in liquid nitrogen using a mortar and pestle, collected into RIPA lysis buffer (50mM Tris-HCl, pH8, 150mM NaCl, 1% NP-40, 0.5% sodium deoxycholate, 0.1% SDS) and incubated on a rotating platform at 4°C for 1h. Lysate was clarified by centrifugation at 16000×g for 10 min and supernatant quantitated by BCA assay (Thermo Scientific). Samples were run on 4–12% NuPAGE gels (Thermo Scientific) and transferred to PVDF membranes (Bio-Rad). Membranes were probed with antibodies against Atrogin-1 (MAFbx; sc-166806, Santa Cruz Biotechnology), MuRF-1 (sc-398608, Santa Cruz Biotechnology) or Hdac4 (#7628, Cell Signaling Technology), with β-actin (ab8227, Abcam) as a loading control. Primary antibodies were visualized by chemiluminescence (Thermo Scientific) after incubation with HRP-conjugated secondary antibodies (GE Healthcare or Santa Cruz Biotechnology).

### Statistical analysis

Data were analyzed using the open source statistical program R (http://www.R-project.org/) by one-way ANOVA with Bonferroni post hoc analyses (p = 0.05). Post hoc analyses were used to explore whether BoNT/A-induced paralysis differed at any time point (day 7, 14, 28) versus naïve controls (* p<0.05; **p<0.01; ***p<0.001). Data were graphed as mean ± SE.

## Results

### BoNT/A induces muscle atrophy and activates myogenic gene expression

**Fig 1:**
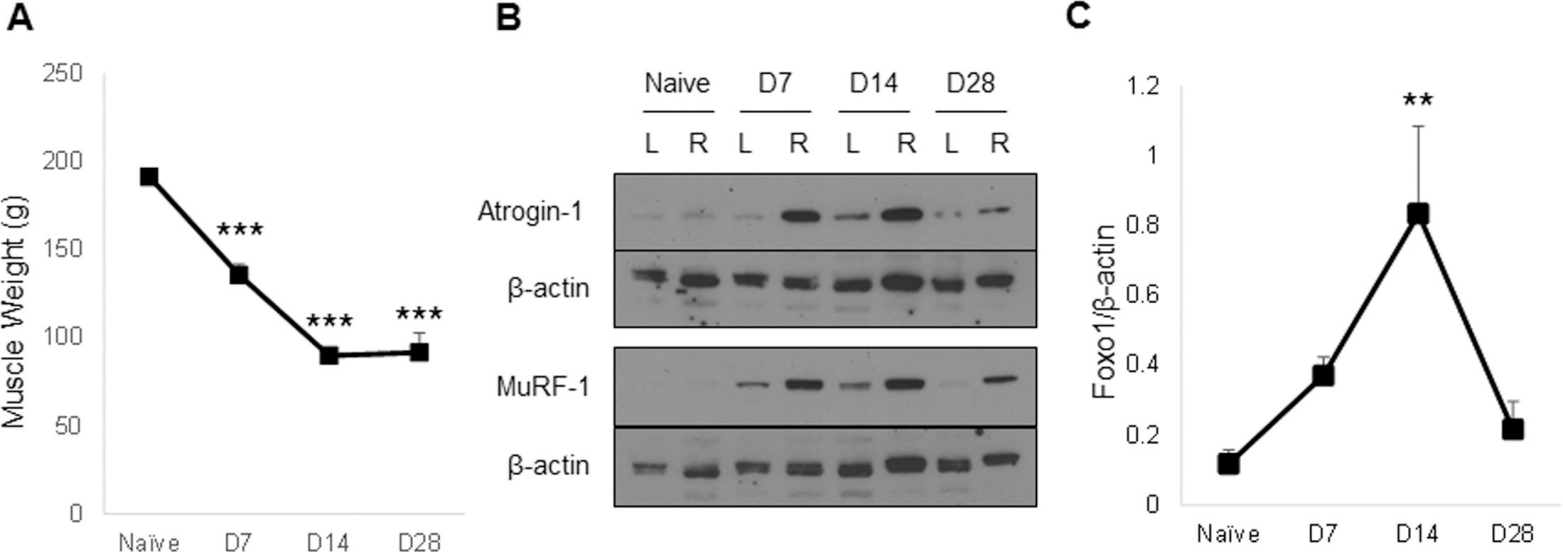
BoNT/A injection of quadriceps leads to muscle atrophy. (A) Muscle weights of right quadriceps dissected from naïve controls and experimental mice at time points shown after single 2.0U BoNT/A injection. (B) Protein levels of Atrogin-1 and MuRF-1 ubiquitin ligases were evaluated by western blotting in whole cell lysates of quadriceps muscle samples of naïve and experimental mice at time points after 2.0U BoNT/A injection. Representative data are shown relative to the β-actin loading control. (C) *Foxo1* mRNA expression was assessed from injected quadriceps muscle samples, at 7, 14 or 28 days after injection with 2.0U BoNT/A and compared to naïve controls (n-4-10/group). Expression levels were quantitated relative to *β-actin*.

We previously reported maximal calf muscle atrophy at 28 days after a single BoNT/A injection [26]. Here we observed significant reduction in muscle weight at 7, 14 and 28 days post BoNT/A treatment, compared with naïve quadriceps (Fig 1A). There was no significant difference in weight of injected muscles between day 14 and 28 (quadriceps masses were 47±1 and 48±6% of naive values, respectively). Upregulation of ubiquitin ligases Atrogin-1 and MuRF-1 in BoNT/A injected muscles was confirmed by Western blot (Fig 1B). At day 14, we observed a significant increase of Forkhead box O1 (*Foxo1*) transcription factor, one of several factors that mediate upregulation of atrophy related ubiquitin ligases (Fig 1C; [59, 60]).

Previous observations of myogenic gene expression following BoNT/A injection have varied, possibly due to differences in injection protocols and muscle groups studied [7, 45, 61, 62]. We did not observe significantly altered expression of nAChRs *Chrna1* and *Chrng* which previously were found to be regulated with denervation and BoNT/A-induced skeletal muscle atrophy [63]; however, there was an increase of the early response gene *Musk* at days 7 and 14 post injection (Table 2). There were also increased expression of genes encoding myogenic regulatory factors *Myod*, *Myog* and *Myf5* at later time points, and enhanced expression of collagen component genes *Col1a1* and *Col3a1* at 28 days compared to the naïve control.

**Table 2:** Relative mRNA expression in quadriceps muscle following 2.0U BoNT/A injection.

		Days after BoNT/A		
	Naïve	Day 7	Day 14	Day 28
*Chrna1*	0.002 ± 0.001	0.007 ± 0.002	0.007 ± 0.002	0.008 ± 0.002
*Chrng*	0.03 ± 0.02	0.65 ± 0.59	0.30 ± 0.06	0.10 ± 0.05
*Musk*	0.06 ± 0.01	1.38 ± 0.31***	1.73 ± 0.27***	0.52 ± 0.13
*Myod*	0.04 ± 0.01	0.12 ± 0.01	1.09 ± 0.35**	0.85 ± 0.27*
*Myog*	0.02 ± 0.00	0.70 ± 0.10	2.70 ± 0.55	5.63 ± 2.22**
*Myf5*	0.002 ± 0.000	0.005 ± 0.001	0.019 ± 0.003***	0.014 ± 0.005*
*Col1a1*	0.04 ± 0.01	0.07 ± 0.03	0.11 ± 0.02	0.40 ± 0.07***
*Col3a1*	0.04 ± 0.01	0.06 ± 0.03	0.06 ± 0.01	0.12 ± 0.02**

Values are means ± SE.

*p<0.05

**p<0.01

***p<0.001 vs. Naïve.

### BoNT/A-induced muscle paralysis differentially alters expression of muscle miRNA

**Fig 2:**
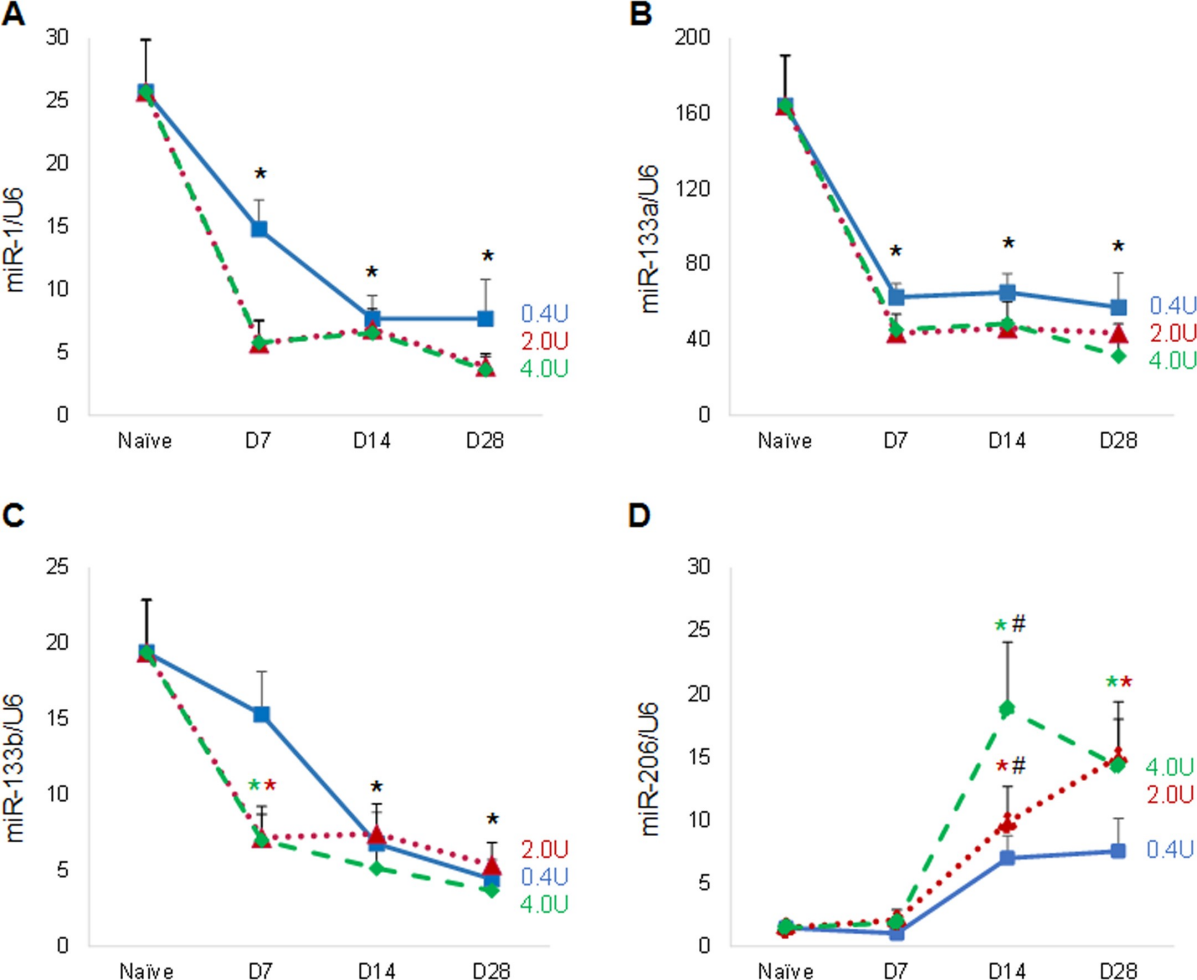
BoNT/A alters muscle miRNA expression. Levels of *miR-1* (A), *miR-133a* (B), *miR-133b* (C) and *miR-206* (D) from naïve and injected quadriceps muscle samples at day 7, 14 and 28 after BoNT/A treatment (n = 3-10/group) were assessed. Results from dosage groups of 0.4 (denoted with blue solid line), 2.0 (red dotted line) and 4.0 (green dashed line) U/100g body weight are shown. Expression was quantitated relative to levels of the small nuclear RNA *U6*. (* p<0.05 vs. naïve; ^#^ p<0.05 vs. 0.4U dose).

Muscle miRNA expression is altered by a variety of muscle atrophy pathologies, including denervation [33, 64]. Here we also observed altered expression of muscle miRNAs following BoNT/A-induced muscle paralysis. The bicistronic partner transcripts *miR-1* (Fig 2A) and *miR-133a* (Fig 2B) demonstrated decreased expression at all BoNT/A doses and time points compared to naïve mice (ranging from -32 to -86% and -60 to -81% respectively). *miR-133b* expression (Fig 2C) was also decreased compared to naïve mice, with the exception of the lowest dose at the earliest time point. In contrast, *miR-206* (Fig 2D), which shares a genomic locus with *miR-133b*, exhibited increased expression at the 2.0 and 4.0U BoNT/A doses at the later collection days (14 and 21) compared to naïve mice (6- to 12-fold elevation). We observed a dose related increase in *miR-206* expression at day 14 but not at other time points. Finally, while the non-muscle specific *miR-29b* has a role in some models of muscle atrophy [65], here expression was 3 orders of magnitude lower than the examined muscle miRNAs, and was not altered by paralysis (S1 Fig).

### BoNT/A-induced muscle paralysis activates known miR-133a/b target genes

**Fig 3:**
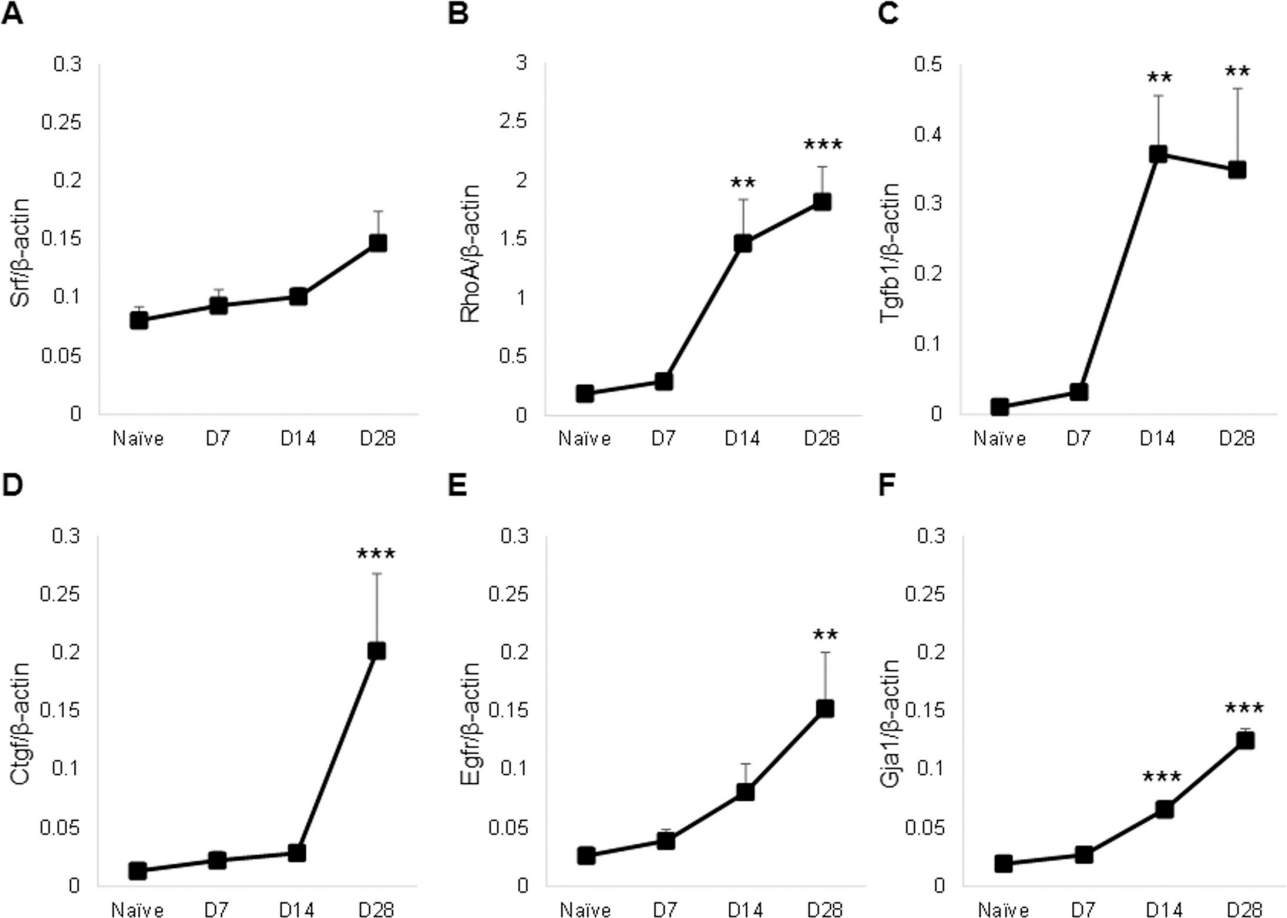
BoNT/A alters expression of known *miR-133a/b* target genes. Levels of *Srf* (A), *RhoA* (B), *Tgfb1* (C), *Ctfg* (D), *Egfr* (E) and *Gja1* (F) mRNA were assessed from naïve and injected quadriceps muscle samples, at 7, 14 or 28 days after injection with 2.0U BoNT/A, and compared to naïve controls (n-4-10/group). Expression levels were normalized relative to *β-actin*.

Contradictory roles for *miR-133* in targeting expression for the promotion of myoblast proliferation, and the repression of myoblast proliferation to drive differentiation [39, 44], suggest that the role of these *miR-133* sequences may be context specific. Levels of expression of validated *miR-133* target genes were thus investigated after injection of quadriceps with 2.0U BoNT/A. While, *miR-133* has been found to regulate *Srf* expression in muscle during proliferation [39], we did not observe significantly altered *Srf* mRNA expression at any time point after BoNT/A injection (Fig 3A). For the GDP-GTP exchange protein RhoA, levels of mRNA were significantly increased at day 14 and 28 following muscle paralysis vs naïve quadriceps (7- and 9-fold elevation, respectively; Fig 3B). Increased expression was also observed for *Tgfb1* at the same time points (>30-fold elevation; Fig 3C). Another growth factor, *Ctgf*, regulated by *miR-133* in muscle cells [55, 56], showed a 15-fold increase in mRNA levels at day 28 after BoNT/A injection compared to the naïve control (Fig 3D). Likewise, there were higher levels of *Egfr* at this timepoint (5-fold increase; Fig 3E). Finally, expression of *Gja1*, a target of *miR-133* [58], was significantly elevated at day 14 and 28 (3- and 6-fold respectively; Fig 3F). Interestingly, *Gja1* was previously also found to be a target of *miR-1*/*206* in several mammalian contexts [66, 67].

### BoNT/A-induced paralysis has differential effects on miR-1/206 target gene expression

**Fig 4:**
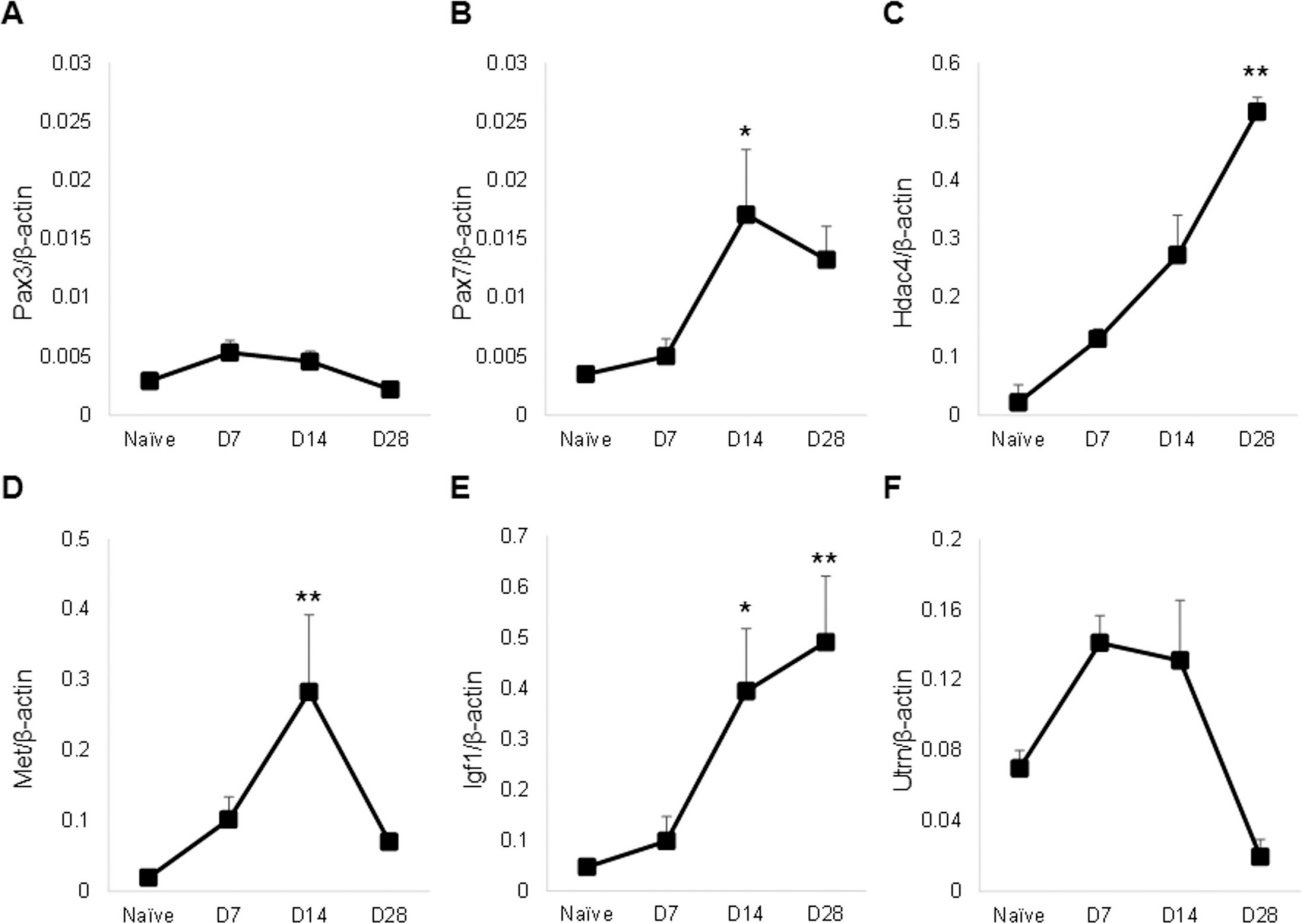
BoNT/A alters expression of known *miR-1/206* target genes. Levels of *Pax3* (A), *Pax7* (B), *Hdac4* (C), *Met* (D), *Igf1* (E) and *Utrn* (F) mRNA were assessed from injected quadriceps muscle samples, at 7, 14 or 28 days after injection with 2.0U BoNT/A, and compared to naïve controls (n-4-10/group). Expression levels were normalized relative to *β-actin*.

As *miR-1* and *miR-206* share a common seed sequence, they have been reported to affect many of the same mRNA target sequences. For example, *Pax3* and *Pax7*, transcription factors that are involved in the transition between proliferative and differentiating satellite cells [68], are both targeted by *miR-1* and *miR-206* [46, 47]. While we did not observe altered *Pax3* mRNA expression (Fig 4A), *Pax7* expression was elevated 5-fold at 14 days after paralysis (Fig 4B). Hdac4, a transcriptional repressor of muscle genes, has also been found to be downregulated in muscle by both *miR-1* and *miR-206* to enable myogenic differentiation [32, 39]. However, in this study *Hdac4* expression was elevated relative to naïve muscles and expression reached statistical significance at day 28 following paralysis (60-fold; Fig 4C). For the proto-oncogene Met, there was a 14-fold increase in expression at day 14 after BoNT/A injection compared to the naïve controls (Fig 4D). There were also increased levels of *Igf-1* at day 14 and 28 after paralysis (8- and 10-fold elevation vs. naïve; Fig 4E). For *miR-206* target, *Utrn*, there were no significant changes in expression after BoNT/A injection (Fig 4F).

### BoNT/A-induced paralysis increases Hdac4 protein and alters Hdac4 target genes

**Fig 5:**
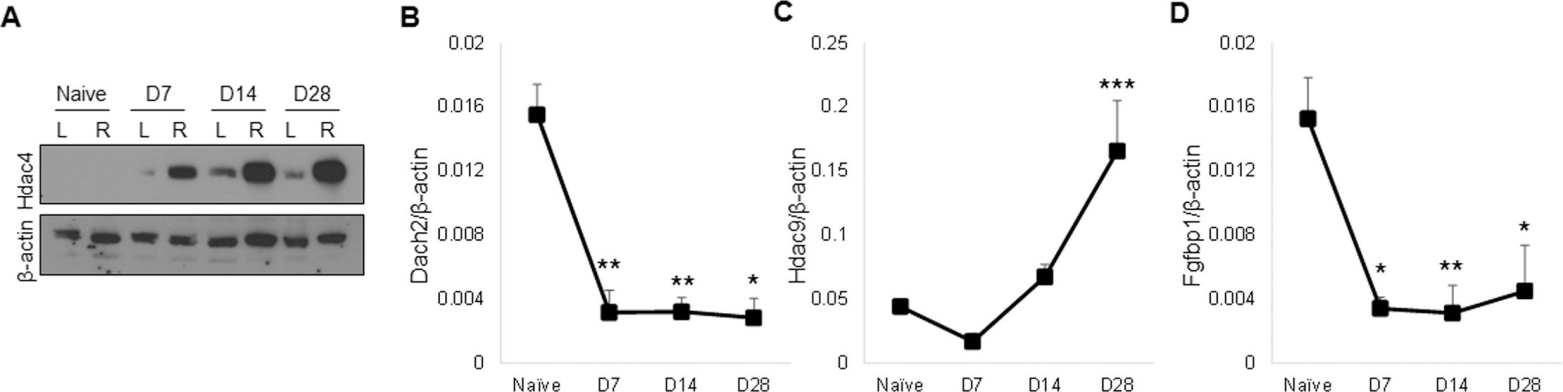
BoNT/A has differential effects on Hdac4 regulated gene expression. (A) Hdac4 protein levels were evaluated by western blotting in whole cell lysates of quadriceps muscle samples at time points after 2.0U BoNT/A injection. Representative data are shown relative to the β-actin loading control (n = 4/time point). Hdac4 regulated mRNA expression was assessed in injected quadriceps muscle samples at 7, 14 or 28 days after injection with 2.0U BoNT/A, and compared to naïve controls (n-4-10/group). Expression levels of *Dach2* (B), *Hdac9* (C) and *Fgfbp1* (D) were quantitated relative to *β-actin*.

Hdac4 is an inhibitor of muscle differentiation [69] and increased levels of this protein have been observed after surgical denervation and in models of muscle atrophy associated with amyotrophic lateral sclerosis (ALS) [12, 13]. Consistent with these findings, increased Hdac4 protein following BoNT/A-induced muscle paralysis was detected in the present study by Western blot (Fig 5A). We then assessed expression of the *Myog* inhibitors Dach2 (dachshund 2) and Hdac9, which are known to be downregulated by Hdac4 during surgical denervation [12, 13]. *Dach2* expression, low in naïve muscles, was significantly lower in paralyzed muscles at all time points after BoNT/A injection (-80%; Fig 5B). *Hdac9* expression was found to be elevated at day 28 post paralysis compared to naïve muscles (Fig 5C). Finally, we observed that expression of *Fgfbp1*, a direct downstream target of Hdac4 in muscle after sciatic nerve crush [32], was reduced in paralyzed quadriceps at all days tested compared to naïve muscle (ranging from -70% to -80%; Fig 5D).

## Discussion

We investigated early molecular responses in muscle following a single intramuscular injection of BoNT/A. BoNT/A-induced muscle paralysis caused rapid and significant muscle atrophy with concurrent changes in expression of key elements of proteasomal protein degradation. At a BoNT/A dose previously associated with severe muscle atrophy (2.0U/100g), we, for the first time, report significantly altered expression of muscle specific miRNAs. Muscle paralysis resulted in significant upregulation of Hdac4 mRNA and protein similar to reports of paralysis following surgical denervation [12, 13]. Taken together, our data suggest that transient muscle paralysis induces altered gene expression that is similar to expression profiles associated with denervation and a variety of neuromuscular pathologies.

With respect to miRNA expression, BoNT/A-induced muscle paralysis precipitated similar alterations as previously observed in alternate neuromuscular atrophy models. Expression of *miR-1*, *miR-133a* and *miR-133b* were all reduced following BoNT/A injection, while *miR-206* was significantly elevated at 14 and 28 days post paralysis. This pattern of altered muscle miRNA expression recapitulates that observed following sciatic nerve transection [33, 70]. Additionally, increased *miR-206* levels have also been associated with onset of neurological symptoms in atrophy models of ALS and SMA (spinal muscular atrophy) [32, 34].

*miR-133a* and *miR-133b* expression were decreased in response to BoNT/A-induced muscle paralysis, with subsequent upregulation of *miR-133* target genes previously implicated in Tgf-β1 mediated hypertrophy and fibrosis of other tissues. For example, in a model of atrial fibrillation, nicotine treated atrial fibroblasts demonstrated reduced levels of *miR-133*, increased expression of *Tgfb1* and its downstream target *Ctgf*, as well as higher levels of collagen production and atrial fibrosis [54]. In a rat model of bladder outlet obstruction, *miR-133* downregulation in bladder smooth muscle cells resulted in similar Tgf-β1 profibrotic signaling, with accumulation of ECM and growth factors consistent with bladder wall hypertrophy and fibrosis [55]. In cardiac myocytes, decreased *miR-133* levels during pathological hypertrophy led to increased Ctgf and collagen synthesis and resultant heart failure [56], and was also associated with increased levels of RhoA, which inhibits axon growth and regeneration [71], and is involved in cardiac hypertrophy [52, 53]. Our observed upregulation of ECM genes are consistent with altered expression 4 weeks post-BoNT/A injection that were identified in a transcript profiling study [7]. It is interesting to speculate that the ECM remodeling and muscle fibrosis underlying BoNT/A-induced changes in muscle mechanical properties [5, 16] may be modulated by downregulation of *miR-133a/b* and a consequent upregulation of ECM genes and growth factors, as observed here.

Based on the literature, we hypothesized a role for *miR-1* in the proteasomal degradation that likely results in NMJ degeneration following BoNT/A-induced muscle paralysis [9, 15]. BoNT/A injection led to differential downregulation of *miR-1* and dose- and time-dependent upregulation of *miR-206*, similar to muscle miRNA expression changes induced by surgical denervation [32, 33, 70]. We also detected upregulation of the downstream targets *Pax7*, *Met*, *Hdac4* and *Igf-1*. As expression of the *miR-206* target *Utrn* (which has not yet been directly associated with *miR-1* regulation) was unchanged in our study, upregulation of these mRNAs was likely attributable to loss of *miR-1* rather than an upregulation of *miR-206*. This interpretation is consistent with the observation that muscle development and regeneration were unaffected by knock-out of the *miR-206/133b* cluster, suggesting that they are expendable, perhaps due to functional overlap with the *miR-1/133a* clusters [72]. In the same context, muscle growth and adaptation were unaffected by manipulation of *miR-206* levels in mice using adeno-associated viral vectors, suggesting *miR-206* may not be a key regulator of these functions in post-natal muscle [73]. However, without further investigation, it is not possible to rule out a role for *miR-206* to counterbalance *miR-1* signaling in fine regulation of the muscle response after BoNT/A injection.

**Fig 6:**
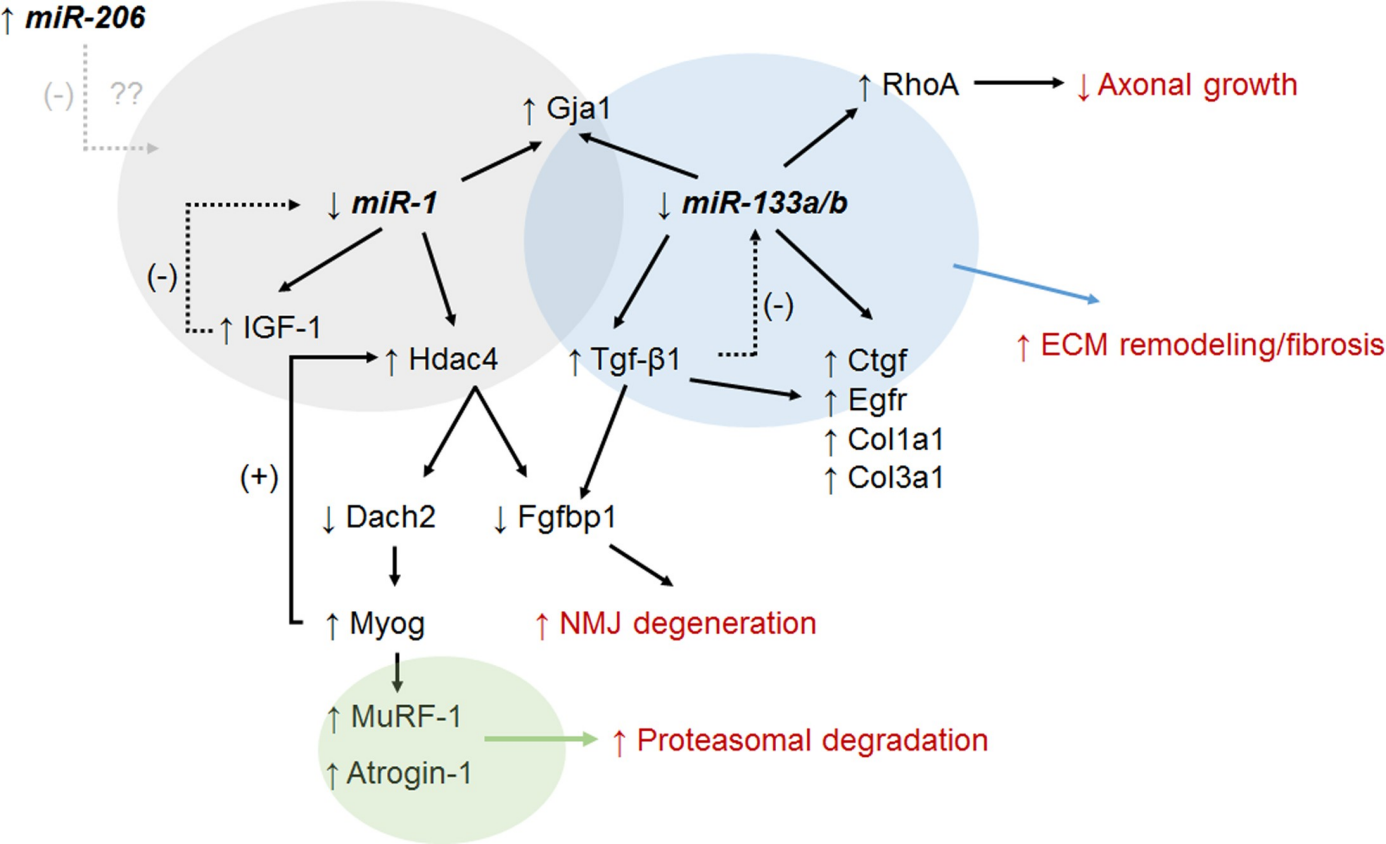
Model of miRNA involvement in muscle response following BoNT/A-induced muscle paralysis. Altered expression of muscle miRNAs potentially impact expression of key mRNA targets that may result in cell and tissue level changes (shown in red). Observed decreases in *miR-133a/b* and *miR-1* levels and associated gene expression changes observed in this study are highlighted in blue and grey ovals respectively. The observed increase in *miR-206* may modulate *miR-1* effects. Observed Hdac4 increase may lead to the increased levels of ubiquitin ligases (green oval) which have been previously implicated in proteasomal degradation.

Contextualizing this miRNA expression pattern with the literature and downstream targets assessed in this study suggests a likely, but complex, role for muscle miRNAs in modulating muscle atrophy induced by transient muscle paralysis (Fig 6). For example, diminished *miR-133a/b* expression is consistent with upregulation of many factors involved in ECM remodeling in other tissues suggesting a potential role for these pathways in muscle structural changes. In turn, downregulation of *miR-1* may be involved in the upregulation of *miR-1/206* targets IGF-1 and Hdac4, and resultant signaling leading to NMJ degeneration and proteasomal degradation. Finally, we speculate that temporal upregulation of *miR-206* may dynamically compete with *miR-1* signaling for fine control of muscle atrophy responses.

The novel observation of upregulation of the differentiation inhibitor Hdac4 following BoNT/A-induced muscle paralysis is consistent with observations in other models of neuromuscular impairment. Enhanced *Hdac4* expression has been reported after sciatic nerve transection, and in neurogenic muscle atrophy mouse models of ALS and NMD (Neuromuscular degeneration) [12], and upregulation of *Hdac4* is associated with faster functional muscle decline and lower muscle re-innervation ability in ALS patients [74]. In addition, our evidence of elevated *Myog* expression, enhanced Atrogin-1 and MuRF-1 protein levels, and downregulated *Dach2* and *Fgfbp1* gene expression strengthen the likelihood that Hdac4 plays a significant role in BoNT/A-induced muscle atrophy, as they are consistent with earlier reports of denervation-induced changes [12–14]. Likewise, our observed decrease in expression of Hdac4 target gene *Fgfbp1* after BoNT/A-induced muscle atrophy, is consistent with Hdac4-Fgfbp1 signaling after denervation [32]. Finally *Hdac9*, another target of Hdac4 [13], was not downregulated in our study, suggesting that while BoNT/A- and denervation- induced atrophy may share similar signaling pathways, they are distinct in some aspects.

Our observations should be weighed in the context of several general limitations. Given their known regulation in a variety of neuromuscular pathologies we focused on quantifying altered canonical muscle miRNA expression; however, other miRNA sequences found to be important in muscle diseases may also have a role in the response of muscle to transient paralysis [75] and our data provide a framework for genome wide analyses of the skeletal muscle response to BoNT/A. Also, we were not able to directly contrast miRNA responses following transient muscle paralysis to other models of muscle dysfunction because the timing, specificity, and extent of miRNA mediated responses are likely to vary across types of neuromuscular injury. Furthermore, our studies largely focused on gene expression changes rather than corresponding protein levels. Altered mRNA levels are not always proportional to protein responses, however, and the relative importance of changes in gene expression therefore cannot be gauged simply from the magnitude of fold change. Additionally, our study investigated short-term molecular responses after BoNT/A induced muscle paralysis without contemporaneous histological documentation of tissue changes. Studies directly relating miRNA/mRNA to histological evidence of muscle atrophy would be of potential relevance, given the widespread use of BoNT/A in the clinic. Finally, our study assessed mRNA and protein patterns in the context of miRNA alterations to survey candidate pathways for future mechanistic assessment, but did not functionally test regulation of these changes by the candidate miRNAs. This approach simplifies the complexity of miRNA regulation, in which feedback loops between signaling proteins such as Igf1 and Tgfb1 affect transcription of canonical muscle miRNAs [76, 77] and miRNA can stimulate as well as inhibit target gene expression [78]. As such, we believe that future studies with genetic models will be required to establish a more direct link between miRNA changes and expression of Hdac4 and to determine the relative significance of other target genes that respond to intramuscular BoNT/A injection.

In summary, we have described molecular changes in the mouse quadriceps following BoNT/A-induced muscle paralysis. We observed, for the first time, differential regulation of muscle miRNAs from the *miR-1/206* and *miR-133* families and increased levels of Hdac4 and downstream mRNA targets following transient muscle paralysis of skeletal muscle. In the context of the literature, our BoNT/A-induced changes appear consistent with a potential role for *miR-133*/Tgf-β1/Ctgf in ECM remodeling and fibrosis previously observed in muscle after injection with this neurotoxin, as well as a role for a *miR-1*/Hdac4/Myog signaling axis in modulating muscle atrophy in this model of muscle paralysis.

## Supporting information

S1 Table Primer sequences used for qRT-PCR of muscle samples. (DOCX)

S1 Fig BoNT/A does not alter muscle *miR-29b* expression. Levels of *miR-29b* from naïve and injected quadriceps muscle samples at day 7, 14 and 28 after BoNT/A treatment (n = 3-10/group) were assessed. Expression was quantitated relative to levels of the small nuclear RNA *U6*.
(TIF)
