# Supplementary material for: Botulinum toxin A-induced muscle paralysis stimulates Hdac4 and differential miRNA expression
Source: PLoS One. 2018 Nov 14;13(11):e0207354. doi: 10.1371/journal.pone.0207354 (PMC6235354; doi:10.1371/journal.pone.0207354)
Supplement: S1 Table — (DOCX) [file pone.0207354.s001.docx]

**S1 Table. Primer Sequences used for qRT-PCR of muscle samples**

| **Target** | **Forward (5′→3′)** | **Reverse (5′→3′)** |
| --- | --- | --- |
| *β-actin* | TCACCCACACTGTGCCCATCTACGA | CAGCGGAACCGCTCATTGCCAATGG |
| *Foxo1* | GCTCTGTGCGCCTAAGTACA | CCGATGGACGGAATGAGAGG |
| *Chrna1* | ATTCCGAGATCTGCCTGTCT | TCTCAAGCAAAAAGTGGTCG |
| *Chrng* | GATGCAATGGTGCGACTATCGC | GCCTCCGGGTCAATGAAGATCC |
| *Musk* | CCAGTACAGAGGGGAGGTGT | TTTCCTTCCATTGCCTGCCA |
| *Myog* | AATGCACTGGAGTTCGGTCC | TCAGTTGGGCATGGTTTCGT |
| *Myod* | GGAGATCCTGCGCAACGCCA | GTAGCCATTCTGCCGCCGGG |
| *Myf5* | AACCAGAGACTCCCCAAGGT | GCTGGACAAGCAATCCAAGC |
| *Col1a1* | AATGGCACGGCTGTGTGCGA | AACGGGTCCCCTTGGGCCTT |
| *Col3a1* | GAGGAATGGGTGGCTATCCG | TCGTCCAGGTCTTCCTGACT |
| *Srf* | GTGATGTATGCCCCCACCTC | CGGGCGGATCATTCACTCTT |
| *Rhoa* | GACCTTCGGAATGACGAGCA | TCTTCCCACGTCTAGCTTGC |
| *Tgfb1* | TTGCTTCAGCTCCACAGAGA | TGGTTGTAGAGGGCAAGGAC |
| *Ctgf* | CCCAACTATGATGCGAGCCA | CGGATGCACTTTTTGCCCTT |
| *Egfr* | TCCTCATTGCCCTCAACACC | CAGAGGATGGGGTTGTTGCT |
| *Gja1* | AGGAGTTCCACCACTTTGGC | AGCGAAAGGCAGACTGTTCA |
| *Pax3* | GTGCTCGCTTTTTCGTCTCG | AAATGACGCAAGGCCGAATG |
| *Pax7* | TGGAAACGGGACAAGCCTAC | GCATGGGTAGATGGCACACT |
| *Hdac4* | CGTTCATCTCTGCAAGGCCA | CTAGCAGCGTCAGTGCCTTA |
| *Met* | GGCTTGTAAGTGCCCGAAGT | TACGTGCTGAACTGCTTGGA |
| *Igf1* | TGGATGCTCTTCAGTTCGTG | GTCTTGGGCATGTCAGTGTG |
| *Utrn* | TGCTAGCCTGGACCATTTTTC | AGGCCTTCGAGAAGATCCAAG |
| *Dach2* | GGTGCTCCGACCCTTAATCC | GAGAGGGAGCAGGAGAAGGA |
| *Hdac9* | CAAAGCTCTCCACCCCTCAG | TCCATCCTTCCGCCTGAGTA |
| *Fgfbp1* | TCTCTGACGCATGGCAAGTT | AGACACTCTTGGCGTTCCTG |
