# Supplementary figures and images for: Botulinum toxin A-induced muscle paralysis stimulates Hdac4 and differential miRNA expression
Source: PLoS One. 2018 Nov 14;13(11):e0207354. doi: 10.1371/journal.pone.0207354 (PMC6235354; doi:10.1371/journal.pone.0207354)

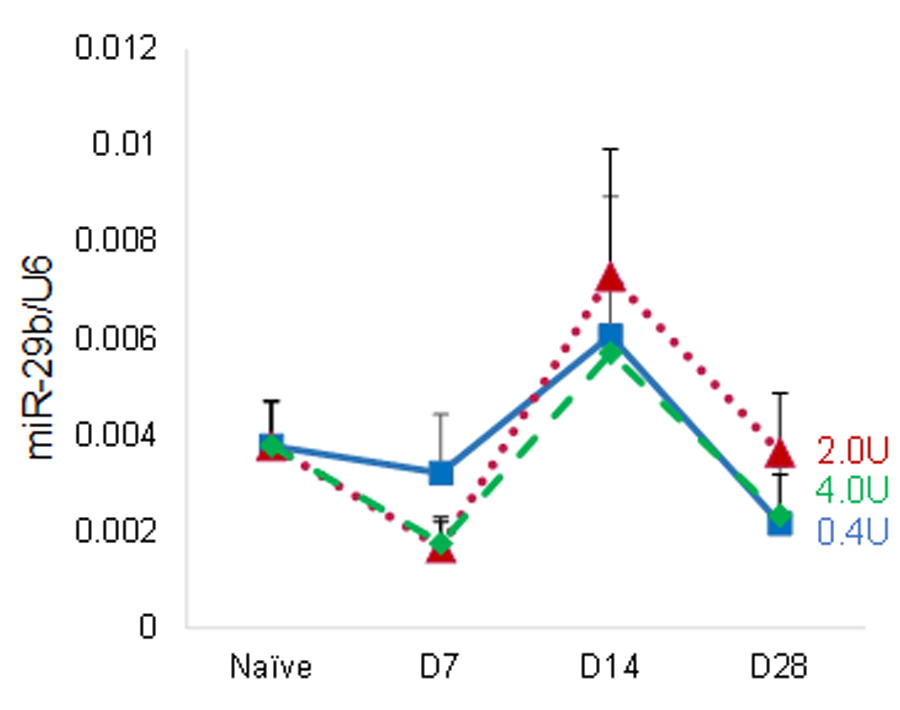

Supplement: S1 Fig — Levels of miR-29b from naïve and injected quadriceps muscle samples at day 7, 14 and 28 after BoNT/A treatment (n = 3-10/group) were assessed. Expression was quantitated relative to levels of the small nuclear RNA U6. (TIF) [file pone.0207354.s002.tif]
